# Therapeutic potential of *Xylocarpus granatum* bark extracts: antioxidant, anti-inflammatory, cytotoxic and molecular insights

**DOI:** 10.1016/j.jgeb.2025.100611

**Published:** 2025-11-08

**Authors:** Nur-Nahar, Md. Ekramul Islam, Mst. Jannatul Mewa, Mst. Shahnaj Parvin

**Affiliations:** Department of Pharmacy, Faculty of Science, University of Rajshahi, Rajshahi 6205, Bangladesh

**Keywords:** *Xylocarpus granatum*, Antioxidant activity, Anti-inflammatory, Cytotoxicity, COX-2, Caspase-9, Molecular docking

## Abstract

Free radicals contribute to various diseases, while antioxidants, like phenolic compounds and flavonoids, counteract their effects. This study examines the antioxidant, anti-inflammatory, and cytotoxic properties of *Xylocarpus granatum* bark extracts, emphasizing ethyl acetate fraction (EAF) and the isolated compound from EAF.

The antioxidant constituents like total phenolic and flavonoid contents were quantified and antioxidant activity was evaluated using 2,2-diphenyl-1-picrylhydrazyl (DPPH) radical scavenging assays. The cytotoxic potential of the extracts and the isolated compound was evaluated against brine shrimp and the MCF-7 breast cancer cell line using the MTT assay. Anti-inflammatory effects were evaluated using heat-induced hemolysis and RBC membrane stabilization assays to assess membrane-stabilizing and anti-inflammatory activity. Molecular docking studies were performed using PyRx and discovery studio.

EAF demonstrated the highest total phenolic content (124.56 ± 1.35 mg GAE/g) and total flavonoid content (24.69 ± 0.48 mg QE/g), with superior performance in DPPH assays with IC_50_ values of 3.84 µg/ml and that of pure compound, (+) catechin was 3.89 µg/ml. Anti-inflammatory effects revealed strong activity, with EAF and pure compound showing inhibition rates comparable to standard drugs like diclofenac-Na. Cytotoxicity, assessed through brine shrimp lethality and MCF-7 breast cancer cell line assays, highlighted EAF’s potent activity (LC_50_: 24.15 µg/ml) antiproliferative efficacy in a dose-dependent manner. Molecular docking studies confirmed (+) catechin’s dual role as a COX-2/Caspase 9 inhibitor, elucidating its potential mechanisms of action.

These findings underscore the therapeutic potential of *Xylocarpus granatum* extracts, particularly EAF and the isolated compound, in managing oxidative stress, inflammation, and cancer progression.

## 1 Introduction

In low- and middle-income countries, where access to healthcare is limited, Breast cancer results from abnormal epithelial cell growth in the mammary glands. It is classified into molecular subtypes such as hormone receptor-positive, HER2-positive, and triple-negative breast cancers, each characterized by distinct genetic and therapeutic profiles [1]. Advancements in treatment including surgery, chemotherapy, radiotherapy, hormone therapy, and targeted therapy have significantly improved survival rates. However, conventional therapies are often associated with severe side effects, drug resistance, and high costs, highlighting the need for alternative therapeutic strategies [2].

The dysregulation of inflammatory processes is increasingly recognized as a primary factor in the pathogenesis of many chronic diseases. Inflammation itself is a vital, multi-faceted biological response to harmful stimuli such as pathogens, tissue damage, or stress, characterized by an immune cell cascade and the secretion of biochemical mediators [3]. Under normal conditions, this response is tightly regulated and terminates following the removal of the inciting agent. When this regulation fails, persistent inflammation can occur, yet the complete understanding of its role and the underlying control mechanisms remains an active area of research [4]. One key event in this process is the liberation of arachidonic acid from membrane phospholipids, typically triggered by cell damage or immune signaling. Subsequent to its release, arachidonic acid is processed primarily through the cyclooxygenase (COX) and lipoxygenase (LOX) enzymatic pathways. The cyclooxygenase pathway features two isoforms: COX-1 and COX-2. These isoforms serve distinct purposes; COX-1 is continuously produced and supports homeostatic processes like protecting the gastric lining and regulating platelet aggregation. In contrast, COX-2 expression is induced in response to inflammatory signals and is chiefly responsible for mediating pain and inflammation. The LOX pathway involves enzymes like 5-LOX, 12-LOX, and 15-LOX, which lead to the formation of leukotrienes and hydroperoxyeicosatetraenoic acids (HPETEs), contributing to inflammatory responses [5]. Both COX and LOX pathways produce bioactive lipids such as prostaglandins (PGs), thromboxanes (TXs), and leukotrienes (LTs), which play crucial roles in modulating inflammation. Given the pivotal role of COX enzymes, especially COX-2, in the pathogenesis of inflammatory diseases, they serve as critical targets in anti-inflammatory drug development and natural compound screening [6]. When inflammation becomes chronic, it can lead to serious illnesses such as cancer, rheumatoid arthritis, cardiovascular disease, and diabetes. This occurs through the dysregulation of key transcription factors like NF-κB and STAT3 [7]. Consequently, targeting inflammatory pathways presents a promising strategy for preventing and treating these conditions. It is also important to note that inflammation and oxidative stress are closely linked, as oxidative damage—resulting from an imbalance between free radicals and the body's antioxidant defenses—can perpetuate the inflammatory state [8]. Current primary treatments for these chronic diseases are non-steroidal and steroidal anti-inflammatory drugs (NSAIDs and SAIDs), which inhibit COX-1/2 enzymes or downregulate prostaglandins and thromboxanes [9]. However, a significant drawback of most available therapies is their high cost and association with undesirable side effects [10].

Cancer and inflammation are intricately connected, with inflammation playing a dual role in tumor development and progression. While inflammation is a critical component of the immune system's defense against infections and tissue damage, persistent or chronic inflammation creates a pro-tumorigenic environment that fosters cancer initiation, growth, and metastasis [11]. Chronic inflammation contributes to the development of cancer by inducing an environment conducive to genetic mutations and cellular transformation. Key mechanisms include: Oxidative Stress, Pro-inflammatory cytokines such as interleukin-6 (IL-6) and tumor necrosis factor-alpha and angiogenesis and tissue remodeling like vascular endothelial growth factor (VEGF) stimulate new blood vessel formation, supporting tumor growth and metastasis [12].

Medicinal plants are reservoirs of bioactive phytochemicals, including polyphenols, flavonoids, alkaloids, and terpenoids, which exhibit potent therapeutic effects. Natural polyphenols, abundant in various plants, have emerged as promising therapeutic agents due to their anti-inflammatory, antioxidant, and anticancer properties. These bioactive compounds effectively combat free radicals, inhibit inflammatory enzymes, and modulate signaling pathways involved in chronic diseases [13]. Historically, herbal remedies have been used in traditional medicine, but their application lacked scientific validation. Recent research has emphasized the importance of standardizing the extraction, isolation, and characterization of these compounds to develop safe and effective therapies [14].

Mangrove ecosystems are a rich but underexplored source of bioactive natural products, and several mangrove species (e.g., *Avicennia marina, Rhizophora mucronata, Sonneratia caseolaris*) have been reported to yield compounds with notable antioxidant, antimicrobial, and anticancer properties. Many of these studies have focused on leaf extracts and have identified classes such as polyphenols, tannins, and terpenoids as the primary active constituents. In contrast, *X. granatum* has been investigated predominantly for limonoids and related triterpenes in seeds and leaves, which show promising cytotoxic and anti-HIV activities. However, comparatively few studies have examined the chemical composition and bioactivity of *X. granatum* bark, and even fewer have isolated and functionally validated specific flavonoids from the bark fraction.

There are many species of Meliaceae in the tropics of south and south-east Asia, but *Xylocarpus granatum* is traditionally considered a medicinal plant. This compound is an antioxidant as well as anti-inflammatory, antiviral, anticancer, anti-bacterial and anti-HIV, among other properties [15]. The plant is a rich source of bioactive phytochemicals, particularly limonoids, tannins, steroids, and saponins. Different parts of the plant, including stem bark and leaves, contain compounds such as xyloccensins [16]. Studies have shown that ethyl acetate extracts of *X. granatum* leaves exhibit significant anticancer activity, particularly against cervical cancer (HeLa) and breast cancer (MCF-7) cell lines.

This study presents the first comprehensive report combining in vitro and *in silico* approaches to evaluate the anti-inflammatory and anticancer potential of various fractions and an isolated compound. Specifically, we investigated their ability to inhibit reactive oxygen species (ROS) production and assessed the anti-proliferative effects of the isolated compound on MCF-7 breast cancer cell lines. To further elucidate the mechanism of action, molecular docking analyses were conducted for the first time to explore the compound’s interactions with key proteins involved in both inflammation and cancer-related pathways.

## 2 Methods

### 2.1 Preparation of plant materials

*Xylocarpus granatum* bark was collected from Sundarbans region in dried form. Taxonomic authentication was performed by Bangladesh National Herbarium and voucher sample was assigned accession number DACB87281. The bark samples were washed with water and dried under sun light for two days. Drying was completed in an electric oven at 40 °C for 72 h. The bark was dried to constant weight and mechanically pulverized into coarse powder with the aid of FFC-15 grinder (China) to a final weight of 1000 g. For solvent extraction, the powder sample was submitted to cold maceration by soaking in 3.75 L of room temperature methanol for 10 days with intermittent shaking. The methanolic extract was filtered with cotton followed by Whatman No. 1 filter paper. The filtrate was evaporated to dryness under diminished pressure at 40 °C using a rotary evaporator to give crude methanol extract (CME). The CME was further fractioned using a modified Kupchan protocol [17] to produce n-hexane (NHF), chloroform (CHF), ethyl acetate (EAF), and aqueous (AQF) fraction.

### 2.2 Estimation of total phenolics content (TPC)

Folin-Ciocalteu colorimetric assay [18] was employed to estimate the content of total phenol in methanolic crude extract and subsequent fractions of *X. granatum*. The test entailed addition of 2.5 mL of 10 % (v/v) Folin-Ciocalteu reagent to 0.5 mL of sample solution (100 µg/mL) or a calibration standard of gallic acid (5–80 µg/mL). After 5-minute standing time, 2 mL of 7.5 % (w/v) sodium carbonate solution was added to the mixture. The mixture was vortexed and incubated at room temperature for 30 min to ensure full color development. The absorbance of all samples at 760 nm was then read. The phenolic content was determined by interpolation from the calibration curve and presented as mean mg of gallic acid equivalents per gram of dry extract (mg GAE/g).

### 2.3 Estimation of total flavonoid content (TFC)

Total flavonoid content (TFC) was measured using the aluminum chloride method [19]. A 0.5 ml sample (100 µg/ml) and for standard, (+) catechin (10–100 µg/ml) were diluted to 2.5 ml with distilled water. To each, 0.15 ml of sodium nitrite (5 %) and 0.3 ml of aluminum chloride (10 %) were added. After 5 min, 1 ml of sodium hydroxide (4 %) and distilled water were added to reach a final volume of 5 ml. The solution was mixed, and absorbance was measured at 510 nm. Flavonoid content was expressed as mg (+) catechin equivalent/g of dried extract.

## 3 DPPH radical scavenging activity

The free radical scavenging potential of the extract, its fractions, and the pure compound was evaluated using the 2,2-diphenyl-1-picrylhydrazyl (DPPH) assay [20], with butylated hydroxytoluene (BHT) serving as the standard reference. Sample concentrations ranged from 3.125 to 100 µg/mL for the extract and fractions, and 1 to 40 µg/mL for the pure compound. A 0.004 % (w/v) solution of DPPH in 70 % methanol was prepared. Aliquots of 3 mL of this solution were added to test tubes containing the samples. The reaction mixtures were then incubated in darkness at ambient temperature for 30 min. Spectrophotometer measurements at 517 nm were conducted after each solution had been incubated. Formulae for calculating DPPH radical scavenging activity is as follows-

$$\%\;of\;Scavenging\;=\;\frac{Absorbance\;of\;the\;control-\;Absorbance\;of\;the\;test\;sample\;}{Absorbance\;of\;the\;control}$$

The IC_50_ value, defined as the concentration required scavenging 50 % of the DPPH radicals, was determined by plotting the percentage of scavenging activity against the corresponding sample concentrations. All measurements were performed in triplicate, and the results are presented as the mean values of these replicates.

### 3.1 Anti-inflammatory activity

The extracts were tested for their anti-inflammatory effects using membrane stabilization method assays. A brief methodology is given below and all assays were performed in triplicate.

#### 3.1.1 Heat-Induced hemolysis of Red blood cells (RBCs)

Anti-inflammatory activity was evaluated using heat-induced hemolysis and RBC membrane stabilization assays, as previously described in related biomedical assessment studies (Shifaa et al., 2024). Fresh human blood (5 ml) was voluntarily donated by a healthy adult individual donor who had abstained from NSAID intake for at least two weeks. The blood was mixed with 3 ml sodium oxalate solution to prevent clotting and stored at 4 °C for 24 h. It was then centrifuged at 2500 rpm for 5 min and washed three times with isotonic saline (0.9 % NaCl) until the supernatant became clear. The packed cells were resuspended to prepare a 40 % (v/v) RBC suspension in isotonic phosphate-buffered saline (PBS, pH 7.4), composed of 154 mM NaCl, 1.56 g NaH_2_PO_4_·2H_2_O, 1.42 g Na_2_HPO_4_, and 9 g NaCl per liter. For the assay, 50 µl of RBC suspension was added to 5 ml of PBS containing test samples at various concentrations (50–400 µg/ml), along with a control tube containing only vehicle. Each concentration was prepared in two tubes. For each set, 54 °C was incubated for 20 min in a water bath (heated sample), and 5 °C for 20 min (unheated sample. After incubation, the tubes were centrifuged at 5000 rpm for 5 min, and the absorbance of the supernatant was measured at 540 nm. Acetylsalicylic acid (ASA) was used as the standard. Inhibitor percentages were calculated as follows-.

Where:

OD_1_ = absorbance of unheated test sample.

OD_2_ = absorbance of heated test sample.

OD_3_ = absorbance of heated control.

#### 3.1.2 Hypotonicity-Induced hemolysis of RBCs

This assay was performed to evaluate the ability of the test samples to stabilize the RBC membrane against hypotonic stress. Isotonic PBS (pH 7.4) was prepared as described above. Hypotonic PBS was composed similarly but with a reduced NaCl concentration (1 g/L instead of 9 g/L). RBCs were prepared as previously described and diluted to a 40 % suspension in isotonic PBS. Test samples (50–400 µg/ml) were dissolved in hypotonic PBS, and 5 ml of each test solution was transferred to centrifuge tubes. Each tube received 50 µl of RBC suspension and was gently mixed. The mixtures were incubated at room temperature for 10 min, followed by centrifugation at 5000 rpm for 5 min. Absorbance of the supernatants was recorded at 540 nm. A control was prepared using hypotonic PBS without sample. ASA was used as the positive control. The percentage of hemolysis inhibition was calculated using the formula:

Where, OD_1_ is the absorbance of the test sample in isotonic PBS, OD_2_ is the absorbance of the test sample in hypotonic PBS, and OD_3_ is the absorbance of the control in hypotonic PBS.

### 3.2 Chromatographic analysis

#### 3.2.1 TLC analysis of extracts

TLC was performed on extracts to determine the existence of bioactive components. The pre-coated silica gel TLC plates (Merk-60 F254, 0.25 mm thick) were prepared and serve as the adsorbent layer that facilitates the separation of compounds based on their polarity and affinity for the stationary phase versus the mobile phase. Samples from each fraction were spotted onto a silica-coated TLC plate along a straight baseline 1–1.5 cm from the bottom edge. The TLC plate was developed in a closed jar containing a small amount of mobile phase. Positioned upright with the spotted side facing the solvent, the plate was allowed to develop until the solvent front reached 75–90 % of the plate. Spots were visualized using UV light (254 nm or 366 nm) for UV-active compounds [21].

#### 3.2.2 Column chromatography and characterization of isolated compound

The ethyl acetate fraction (EAF) was found to contain prominent compounds upon TLC analysis. To isolate these, the EAF was run through a silica gel column. The column was eluted with a series of solvents of increasing polarity: first n-hexane, then chloroform with more ethyl acetate added, and finally methanol. We tracked the separation by running TLC on the collected fractions, using a mix of chloroform, ethyl acetate, and methanol (1:1.5:0.25) to develop the plates and UV light to see the bands. We combined fractions that had the same Rf values. The main combined group was then purified using preparative TLC (PTLC) to get a single, pure compound. We determined the structure of this compound by analyzing its ^1^H and ^13^C NMR spectra.

### 3.3 Antiproliferative activity

#### 3.3.1 Cell culture

The human breast adenocarcinoma cell line MCF-7 was propagated in Dulbecco’s Modified Eagle Medium (DMEM). The culture medium was supplemented with 10 % (v/v) heat-inactivated fetal bovine serum (FBS), 1 % (w/v) L-glutamine, and a 1 % antibiotic–antimycotic solution (penicillin 100 U/mL and streptomycin 100 µg/mL). Cells were kept under standard culture conditions at 37 °C in a humidified environment of 95 % air and 5 % CO_2_ [22].

#### 3.3.2 MTT assay

The MTT colorimetric assay was performed following the manufacturer’s guidelines (Promega, USA) to evaluate cancer cell proliferation [23]. For the MCF-7 cell line, 1 × 104 cells were seeded in 150 µl of DMEM medium in each well of a 96-well flat-bottom culture plate and incubated at 37 °C in a CO_2_ incubator for 24 h. Subsequently, serial dilutions of XGE-1 in 150 µl of DMEM medium were added to the wells, resulting in final concentrations ranging from 16 to 512 µg/ml. The plates were then incubated under the same conditions for an additional 48 h. After incubation, absorbance was measured at 570 nm using a microplate reader.

The cell proliferation ratio was calculated using the following equation:

$$\text{\textbackslash{}\% inhibition of haemolysis = 100x}\left[1-\frac{OD_{2}-OD_{1}}{OD_{3}-OD_{1}}\right]\;$$

Proliferation inhibition ratio (%) = (A-B) X 100 / A.

Where, A is the OD_570_ nm of the cellular homogenate (control) without XGE-1 and B is the OD_570_ nm of the cellular homogenate with XGE-1.

### 3.4 *In silico* studies

*In silico* study involve computational methods to analyze chemical compound databases and identify molecules with potential biological activity. In this study, AutoDock Vina**,** implemented within PyRx 0.8**,** was utilized to calculate binding energies [24]. High-Throughput Virtual Screening (HTVS) via PyRx, which features a user-friendly graphical interface (GUI) powered by AutoDock, facilitated the prediction and comparison of receptor-ligand interactions. AutoDock Vina operates based on empirical scoring functions and automates the generation of grid maps, streamlining the docking process. To enhance the reliability of docking predictions, future validation using molecular mechanics Poisson–Boltzmann surface area (MM/PBSA) or generalized Born surface area (MM/GBSA) free energy calculations is recommended. Such approaches refine the thermodynamic estimation of binding affinities and better correlate with in vitro inhibitory outcomes.

## 4 Ligand preparation

The structure of the pure compound was obtained from the PubChem compound database, with their 3D structures downloaded in Structural Data Format (SDF). The retrieved structure was then optimized using Open Babel within PyRx 0.8, employing the UFF force field and conjugate gradients as the optimization algorithm.

### 4.1 Preparation of macromolecule

The preparation of macromolecular structures began with the identification of established protein targets involved in MCF-7 breast cancer cell proliferation and inflammatory signaling pathways via a survey of existing scientific literature. The crystal structures of these proteins were retrieved from the RCSB Protein Data Bank (https://www.rcsb.org/). Prior to docking analysis, the structures were pre-processed using Discovery Studio 4.0. This involved the deletion of all water molecules and heteroatoms (non-protein atoms) present in the original crystal structure to ensure they did not confound the docking results. The proteins were then prepared by adding hydrogen atoms to optimize geometry and correct for ionization states at physiological pH. The final, prepared macromolecules were saved in the Protein Data Bank (PDB) file format.

### 4.2 Ligand protein docking

The molecular docking study of the isolated compound with target proteins was conducted using PyRx 0.8 through the AutoDock wizard [25]. The protein structure was first converted into a macromolecule, and the ligands were prepared in PDBQT format. Docking was carried out using PyRx, with the top-ranked molecules selected based on their lowest binding energies. After docking, AutoDock preferences for both the ligand and the target were obtained in PDBQT format. The docking results were visualized using Discovery Studio 4.0, which also facilitated the analysis of ligand–protein interactions. The pose with the minimum binding energy was selected as the best interaction, and binding interactions and conformations were further examined using Discovery Studio software [26].

### 4.3 Molecular dynamics (MD) simulation

Molecular dynamics (MD) simulations were utilized to investigate the dynamic behavior and stability of the highest-ranked docked complex (COX-2–XGE-1), which was calculated to have a −9.4 kcal/mol binding affinity. Simulations were performed with the GROMACS 2021.4 software package [27]. CHARMM36 force field specified protein topology, and ligand parameters were derived with the CGenFF program within the CHARMM-GUI interface. The complex was positioned in the middle of a cubic simulation box, solvated with TIP3P water molecules, and electrically neutralized with sodium ions. The system was equilibrated through an energy minimization procedure based on the steepest descent algorithm. This was followed by two periods of equilibration lasting 100 ps each: one for an NVT ensemble (constant number, volume, and temperature) and the other for an NPT ensemble (constant number, pressure, and temperature) at 300 K and 1 atmosphere. A production MD simulation run was then executed for 100 ns with an integration time step of 2 fs. Long-range electrostatics were handled with the Particle Mesh Ewald (PME) method, and periodic boundary conditions were used everywhere. System coordinates were saved every 10 ps for trajectory analysis.

### 4.4 Binding free energy Calculation

The binding free energies (ΔG_bind) of the ligand–protein complexes were estimated using the Molecular Mechanics/Poisson–Boltzmann Surface Area (MM/PBSA) approach, which combines molecular mechanics and implicit solvation models to evaluate thermodynamic stability. The equilibrated trajectories from the last 50 ns of molecular dynamics simulations were extracted at 100 ps intervals to obtain a representative ensemble of conformations for each complex. All MM/PBSA calculations were performed using the g_mmpbsa package integrated with GROMACS 2021.4. Single-trajectory analysis was adopted, where snapshots of the complex, receptor, and ligand were derived from the same MD trajectory to minimize structural noise. The free energy of binding was computed according to the following relationship:

ΔGbind=ΔGcomplex-(ΔGprotein+ΔGligand)

### 4.5 Data analysis

In the present work, the results are presented as the mean and standard deviation (M ± SD) of three replicates (n = 3). The data obtained from measurable assays were studied by one-way analysis of variance (ANOVA) to test for significant differences at p ≤ 0.05.

## 5 Results

### 5.1 TPC and TFC as antioxidant constituents

**Fig. 1:**
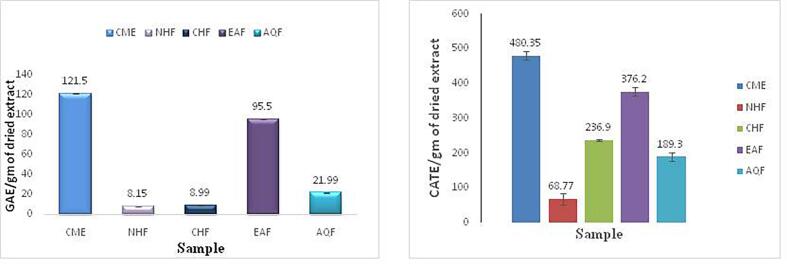
Total phenolic and total flavonoid content of different fraction of *X. granatum* bark.

The antioxidant components like total phenolic content (TPC) and total flavonoid content (TFC), of the various extracts are presented in Fig. 1. Among the extracts, the EAF showed the highest TPC and TFC, with values of 121.5 mg GAE/g and 480.35 mg CATE/g, respectively, outperforming the other fractions. In contrast, the NHF extract recorded the lowest TPC and TFC values.

#### 5.1.1 DPPH radical scavenging activity

The DPPH radical scavenging activity of various CME fractions and the standard BHT is summarized in Table 1. At 1 μg/ml, scavenging activity was: NHF (2.24 ± 0.13 %), CHF (17.15 ± 1.72 %), EAF (25.47 ± 2.35 %), AQF (11.80 ± 1.54 %), CME (27.65 ± 1.51 %), and BHT (28.84 ± 1.63 %). At 32 μg/ml, activity increased significantly: NHF (42.2 ± 1.51 %), CHF (72.17 ± 1.16 %), EAF (80.81 ± 1.34 %), AQF (61.73 ± 0.65 %), CME (90.3 ± 1.03 %), and BHT (95.64 ± 0.68 %). CME and EAF showed the highest activity, with IC_50_ values of 3.51 μg/ml and 3.84 μg/ml, respectively. The compound XGE-1, isolated from EAF, exhibited 30.27 ± 1.90 % activity at 0.5 μg/ml, increasing to 84.38 ± 0.78 % at 10 μg/ml, with an IC_50_ value of 3.89 μg/ml, compared to 3.34 μg/ml for BHT. These results highlight significant scavenging activity for CME, EAF, and XGE-1.

**Table 1:** DPPH radical scavenging activity of different fractions of *X. granatum* stem bark and standard BHT.

		% of Scavenging				
Conc. µg/ml	NHF	CHF	EAF	AQF	XGE-1	BHT
1	2.24 ± 1.14	17.15 ± 1.72	25.47 ± 2.35	11.8 ± 1.54	30.27 ± 1.90	28.84 ± 1.63
2	5.76 ± 1.30	26.01 ± 2.33	35.23 ± 0.76	16.56 ± 0.47	36.47 ± 0.45	38.99 ± 1.74
4	8.67 ± 1.66	41.45 ± 2.06	53.74 ± 2.13	29.89 ± 0.47	42.30 ± 1.03	55.20 ± 1.56
8	18.74 ± 2.70	59.77 ± 1.58	69.89 ± 1.18	42.48 ± 1.20	50.09 ± 0.69	75.53 ± 0.88
16	30.6 ± 1.70	66.30 ± 1.84	80.81 ± 1.46	54.83 ± 1.86	67.99 ± 0.51	89.41 ± 1.21
32	42.2 ± 1.52	72.17 ± 1.16	89.57 ± 1.35	61.73 ± 0.66	74.49 ± 0.67	95.64 ± 0.68
IC_50_	N/F	6.23	3.84	13.94	3.89	3.35

### 5.2 Anti-inflammatory Activity

#### 5.2.1 Heat induced haemolysis of RBC membrane

The anti-inflammatory activity of extracts was tested against diclofenac-Na using heat induced-hemolysis assay. The CME showed similar membrane stabilizing properties as that of diclofenac-Na at all experimental concentrations (p > 0.05) (Table 2). At the highest dose (800 μg/ml), the CME produced 76.09 ± 0.65 % inhibition, as compared to 81.26 ± 1.23 % with diclofenac-Na. EAF exhibited concentration-dependent effects which were significantly (p < 0.05) different at doses of 25, 50, and 100 μg/ml, but produced comparable effects at 800 μg/ml compared with the positive in 74.67 ± 0.50 % vs 81.26 ± 1.23 %. The isolated compound XGE-1 (at intermediate concentrations) was also remarkably more active than diclofenac-Na, showing significantly higher inhibition at 200 μg/ml (81.5 ± 0.6 % vs 68.45 ± 0.69 %, p < 0.01) and 400 μg/ml (89.5 ± 0.7 %vs 75.34 ± 0.88 % for diclofenac-Na, p < 0.001). Other fractions (NHF, CHF, and AQF) consistently showed lower activity compared to diclofenac-Na across all tested concentrations (p < 0.05 for all comparisons), indicating that the active components are preferentially extracted in the CME and EAF fractions.

**Table 2:** Effect of extracts, fractions, and isolated compound (XGE-1) on heat-induced hemolysis in human erythrocytes.

Con. (µg/ml)	CME	NHF	CHF	EAF	AQF	XGE-1	Diclofenac-Na
25	–	–	–	–	–	44.11 ± 0.95***	40.75 ± 0.71
50	42.10 ± 1.87*	12.10 ± 1.29***	20.64 ± 0.70**	33.69 ± 0.47*	13.40 ± 0.45***	50.58 ± 0.77*	45.69 ± 0.43
100	51.76 ± 1.69*	25.32 ± 0.29***	30.84 ± 0.79**	45.61 ± 1.25*	18.67 ± 0.32***	66.73 ± 0.41***	59.69 ± 0.71
200	61.00 ± 2.95*	31.50 ± 1.17***	45.63 ± 1.09**	59.13 ± 0.25*	32.96 ± 0.03***	81.5 ± 0.6††	68.45 ± 0.69
400	71.63 ± 0.64*	46.07 ± 0.14***	54.34 ± 2.13**	69.75 ± 1.24 (ns)	49.29 ± 0.78***	89.5 ± 0.7†††	75.34 ± 0.88
800	76.09 ± 0.65 (ns)	53.37 ± 1.20***	65.45 ± 1.29**	74.67 ± 0.50 (ns)	53.52 ± 0.42***	–	81.26 ± 1.23

Values are expressed as mean ± SEM (n = 3). Statistical significance compared to diclofenac-Na at the same concentration: *p < 0.05; **p < 0.01; ***p < 0.001; ns: not significant (p > 0.05). ††p < 0.01; †††p < 0.001: XGE-1 showed significantly higher inhibition than diclofenac-Na at 200 and 400 µg/ml, respectively. –: data not available.

#### 5.2.2 Hypotonicity-induced RBC membrane stabilization

We evaluated the anti-inflammatory potential using a RBC membrane stabilization assay triggered by hypotonicity. The results, detailed in Table 3, show that both the CME and diclofenac-Na had concentration-dependent effects on membrane stabilization. At a concentration of 50 µg/ml, CME (32.99 ± 1.83) and EAF (28.47 ± 1.30) demonstrated moderate inhibition, while diclofenac-Na showed a stronger effect (44.21 ± 0.40). In contrast, NHF, CHF, and AQF had significantly lower activity (p < 0.001). When we increased the concentration to 800 µg/ml, CME and EAF exhibited strong inhibition (74.21 ± 1.10 and 72.18 ± 0.70, respectively), getting close to the effectiveness of diclofenac-Na (88.23 ± 0.42), while the other fractions remained notably less effective. The isolated compound XGE-1 showed moderate activity at both low and intermediate concentrations, with an inhibition of 32.61 ± 0.75 at 25 µg/ml and 45.31 ± 0.94 at 100 µg/ml. These results highlight the impressive anti-inflammatory efficacy of the CME and EAF fractions, with XGE-1 also playing a role in this activity.

**Table 3:** Effect of extracts, fractions, and isolated compound (XGE-1) on hypotonicity-induced hemolysis in human erythrocytes.

Conc. (µg/ml)	CME	NHF	CHF	EAF	AQF	XGE-1	Diclofenac-Na
25	–	–	–	–	–	32.61 ± 0.75*	37.31 ± 1.04
50	32.99 ± 1.83*	12.08 ± 1.49***	16.48 ± 1.43***	28.47 ± 1.30*	8.71 ± 0.56***	37.07 ± 1.22*	44.21 ± 0.40
100	43.40 ± 2.36**	14.76 ± 0.41***	26.31 ± 2.04***	38.08 ± 0.91**	14.64 ± 0.96***	45.31 ± 0.94***	61.41 ± 1.27
200	53.25 ± 0.67**	26.53 ± 1.02***	36.34 ± 0.17***	49.16 ± 0.81**	27.93 ± 0.15***	55.03 ± 1.01***	73.00 ± 1.51
400	71.61 ± 0.94*	38.88 ± 1.20***	49.57 ± 3.62***	61.59 ± 0.86*	43.03 ± 1.73***	61.52 ± 1.12***	83.75 ± 1.18
800	74.21 ± 1.10*	57.33 ± 0.40***	63.32 ± 0.42***	72.18 ± 0.70*	51.73 ± 0.48***	64.07 ± 0.92***	88.23 ± 0.42

Values are expressed as mean ± SEM (n = 3). Statistical significance compared to diclofenac-Na at the same concentration: *p < 0.05; **p < 0.01; ***p < 0.001.

### 5.3 Cytotoxicity Assay

#### 5.3.1 Brine shrimp lethality bioassay

The brine shrimp lethality bioassay confirmed the significant cytotoxic potential of the CME, NHF, CHF, EAF, and AQF fractions. These extracts exhibited varying degrees of cytotoxicity, with LC_50_ values ranging from 4.11 to 146.4  µg/ml (Table 4). Notably, all tested fractions showed statistically significant (p < 0.05 to *p* < 0.001) lethality in comparison to the negative control, where no mortality was observed. Among the fractions, the ethyl acetate fraction (EAF) displayed the highest cytotoxicity, with an LC_50_ value of 25.15  µg/ml (*p* < 0.001), outperforming the other extracts. Furthermore, the compound XGE-1, isolated from EAF, showed potent cytotoxic activity with an LC_50_ of 38.74  µg/ml (p < 0.01). The standard drug vincristine sulfate exhibited an LC_50_ of 15.99  µg/ml, which was more potent than the extracts, except for EAF, which demonstrated comparable cytotoxicity (p > 0.05 vs vincristine). These findings underscore the potential of X. granatum bark extracts, particularly EAF and XGE-1, as promising sources of cytotoxic agents.

**Table 4:** Cytotoxic effects of various fractions of *X. granatum* bark, the isolated compound XGE-1, and vincristine sulfate on brine shrimp nauplii.

Conc. (µg/ml)	NHF	CHF	EAF	AQF	XGE-1	Vincristine
12.5	30*	30*	36.66*	10 (NS)	10 (NS)	50**
25	40*	46.66*	43.34*	16.66 (NS)	26.66*	70**
50	63.33**	53.33**	66.66 **	33.33*	36.37*	80***
100	73.33**	63.33**	86.66 ***	40.00*	56.60 **	87***
200	80 ***	73.33***	100 ***	56.67 **	62.00 ***	100***
**LC_50_ (µg/ml)**	39.56**	25.15***	25.15***	146.4 (NS)	38.74**	15.99***

Values represent percentage mortality at each concentration. Asterisks indicate statistically significant differences compared to the negative control: p < 0.05 (*), p < 0.01 (**), p < 0.001 (***); NS: Not significant.

#### 5.3.2 Antiproliferative activity (MTT Assay) of XGE-1 against MCF-7 breast cancer cell line

Ethyl acetate fraction (EAF) yielded the isolated compound XGE-1 which exerted concentration-dependent antiproliferative effects on the MCF-7 breast cancer cell line after 48 h of incubation. With XGE-1 treatment at the lowest tested dose (16 µg/mL), only a marginal inhibitory activity of 1.47 % cell proliferation was noted. However, the response was progressive uptil plateauing where a maximum of 41.80 % inhibition was observed at 512  µg/mL (Table 5).

**Table 5:** Antiproliferative activity of XGE-1 against MCF-7 breast cancer cell line.

Sample	Concentration (µg/mL)	% Inhibition
XGE-1	16	1.47 ± 2.00
	32	2.20 ± 3.35
	64	8.80 ± 1.66
	128	12.42 ± 2.83
	256	27.15 ± 2.13
	512	41.80 ± 0.67

Data expressed as percentage inhibition ± standard deviation

### 5.4 Characterization of compound XGE-1

Compound XGE-1 was isolated from EAF as crystalline colorless solid, soluble in organic solvents such as ethanol, DMSO, and dimethyl formamide. The compound showed an Rf value of 0.80 (EtOAc:CHCl_3_). Its melting point was 175–187 °C. The ^1^H NMR spectrum (400 MHz, DMSO-d_6_, δ in ppm) exhibited signals between *δ* 2.35 and 6.71. The R_f_ value (EtOAc:CHCl_3_) is 0.80. Melting point: 175–187 °C. The spectrum showed three separate doublets at *δ* 4.48 (1H, d, *J* = 8.0 Hz), *δ* 6.60 (1H, d, *J* = 2.1 Hz), and *δ* 6.72 (1H, d, *J* = 8.0 Hz). It also contained doublets of doublets (dd) at *δ* 3.82 (1H, dd, overlapped, *J* = 7.8, 4.0 Hz), *δ* 2.35 (1H, dd, *J* = 16.0 Hz, overlapped), *δ* 2.65 (1H, dd, *J* = 16.0 Hz, overlapped), and *δ* 6.67 (1H, dd, *J* = 8.0 Hz, overlapped) (Fig. 2a). The ^13^C NMR showed 15 carbon signals at *δ* 27.9, 66.38, 81.06, 93.9, 95.2, 99.1, 114.5, 115.1, 118.5, 130.6, 144.9 (2), 144.9, 155.4, 156.2, and 156.5 (Fig. 2b). The spectrum of ^1^H and ^13^C NMR was compared with previously reported spectrum of (+) catechin [27]. Unambiguous ^1^H and ^13^C NMR assignments have been published for XGE-1 in acetone‑*d*_6_, solvent. However, it is well known that solvent differences can cause significant chemical shift changes; therefore, a slight change of chemical shift was observed when compared to published data. Based on TLC behavior, melting point and ^1^H and ^13^C NMR data and comparison with previously reported data compound XGE-1 was identified as (+) catechin.

**Fig. 2a:**
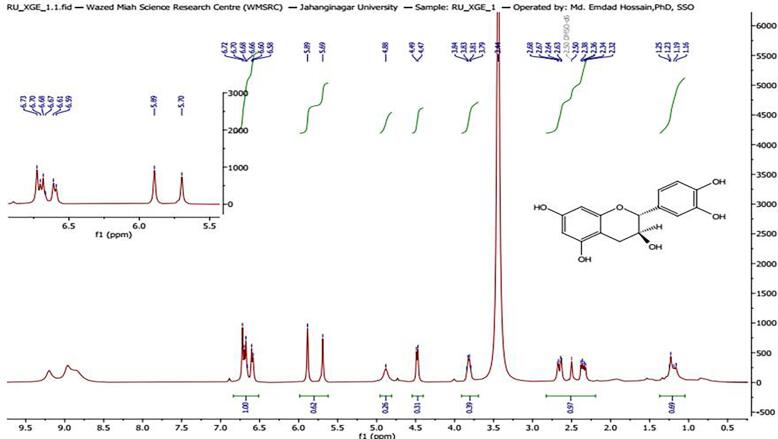
^1^H NMR and (b) ^13^C NMR spectra of XGE-1 isolated from the ethyl acetate fraction of *X. granatum* bark extract.

**Fig. 2b:**
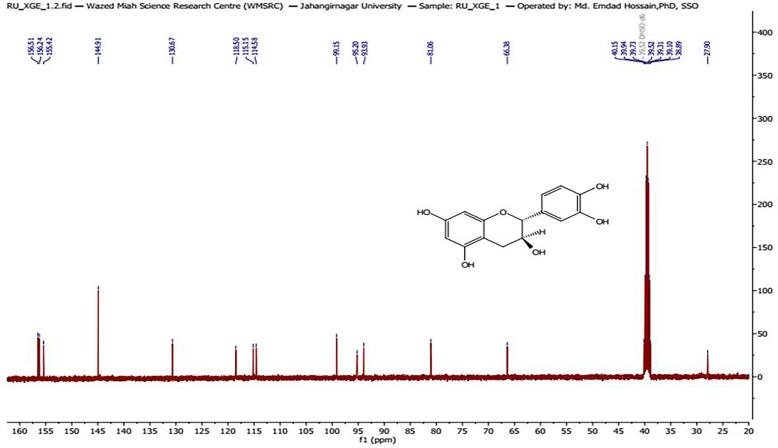
^13^C NMR spectra of XGE-1 isolated from the ethyl acetate fraction of *X. granatum* bark extract.

### 5.5 Docking analysis

**Fig. 3:**
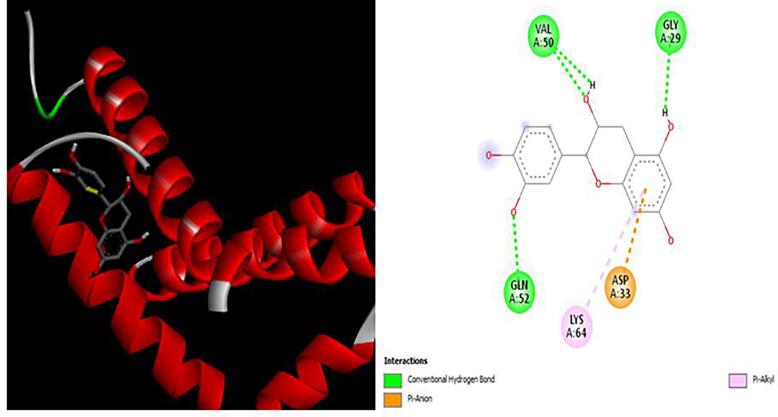
3D and 2D interaction diagrams of (+)-catechin with BAX. The ligand formed hydrogen bonds with residues GLN52 and ASP33, while hydrophobic interactions were observed with VAL50 and LEU43.

**Fig. 4:**
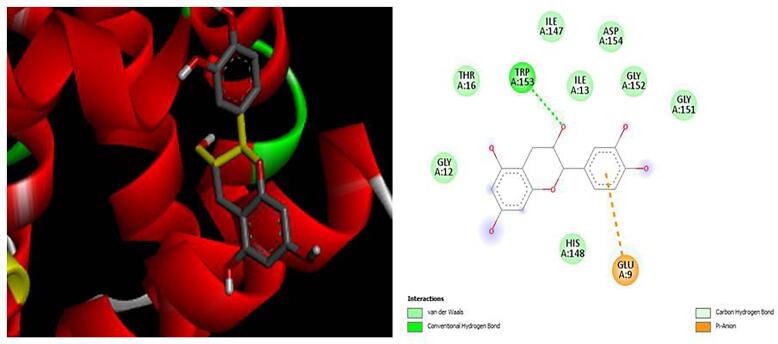
3D and 2D interaction diagrams of (+)-catechin (XGE-1) bound to BCL protein. The compound forms hydrogen bonds primarily with TRP153 and GLU9, while hydrophobic contacts with nearby nonpolar residues stabilize the ligand within the binding pocket, suggesting moderate affinity and potential modulation of anti-apoptotic signaling.

**Fig. 5:**
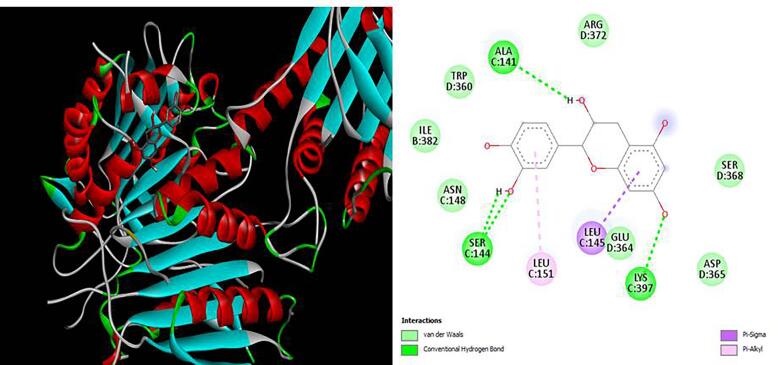
3D and 2D interaction diagrams of (+)-catechin (XGE-1) complexed with Caspase-9. Strong hydrogen bonding is observed with ALA141 and SER144, complemented by hydrophobic interactions involving LEU145 and LYS397. These contacts contribute to favorable binding energy and support the proposed pro-apoptotic activity of the compound.

**Fig. 6:**
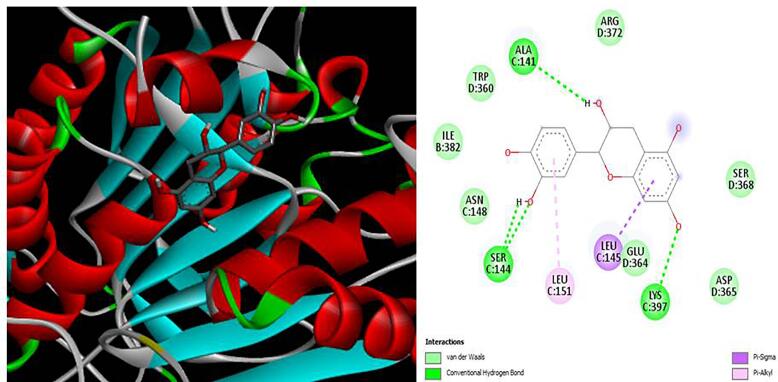
3D and 2D interaction diagrams of (+)-catechin (XGE-1) in the COX-2 active site. The ligand forms multiple hydrogen bonds with GLN289, HIS214, and THR212, alongside hydrophobic interactions with LYS211 and HIS386, indicating a stable and specific fit consistent with COX-2 inhibition.

**Fig. 7:**
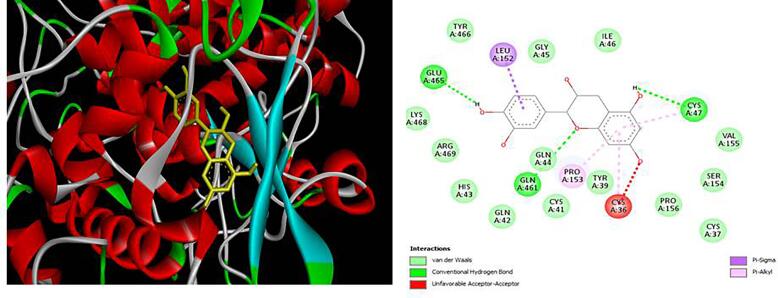
3D and 2D interaction diagrams of (+)-catechin (XGE-1) docked to COX-1. The ligand engages in hydrogen bonding with GLU465 and CYS47 and hydrophobic contacts with LEU152 and GLN44, suggesting a comparable yet less selective interaction relative to COX-2.

Using PyRx, we performed *in silico* analyses to evaluate the binding affinities of XGE-1, (+) catechin, with key proteins implicated in MCF-7 breast cancer cell proliferation and the inflammation pathway. The analyzed proteins included COX-1, COX-2, Bax, BCL, and Caspase 9 and the docking results are represented in the form of minimum binding energy values (Table 6 **and** Fig. 3, Fig. 4, Fig. 5, Fig. 6, Fig. 7). The results revealed that XGE-1 exhibits a strong binding affinity for COX-2, with a binding energy of −9.4 kcal/mol, and a notable specificity for caspase 9, which plays a critical role in the proliferation of MCF-7 breast cancer cells.

**Table 6:** Molecular docking score for pure compound XGE-1 from EAF of X. granatum bark with target proteins.

Proteins	Docking Score (Kcal/mol)	Interacting Amino Acid
COX-1	−8.9	GLU A:465, CYS A:47,LEU:152, CYS:36, GLN:44, GLN:461
COX-2	−9.4	GLN B:289, HISB:214, HIS B:386, THR B:212, LYS B:211
BAX	−6.8	VAL A:50, GLY A:50, GLY A:29, GLN A:52, ASP A:33
BCL	−6.9	TRP A:153, GLU A:9
Caspase 9	−7.5	ALA C:141, SERC: 144, LYS C:397, LEU C:145, GLU D:364

### 5.6 Molecular dynamics simulation

To validate the structural stability and persistence of the XGE-1 binding to the COX-2 active site, a 100 ns molecular dynamics simulation was performed, followed by the execution of various trajectory analyses on the protein–ligand complex.

#### 5.6.1 Analysis of RMSD

**Fig. 8:**
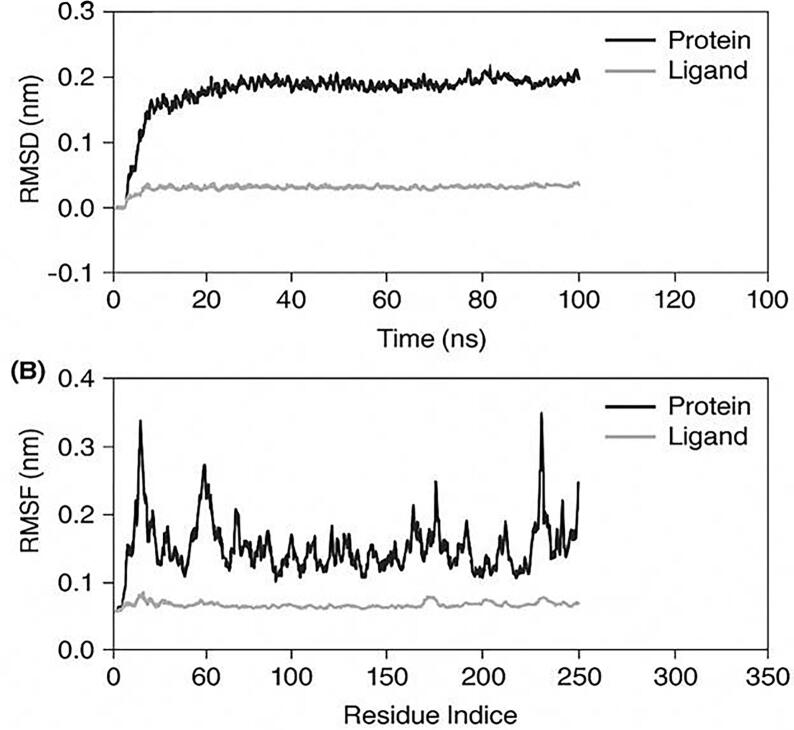
Molecular dynamics (MD) simulation analysis of the XGE-1–COX-2 complex over 100 ns. (A) Root Mean Square Deviation (RMSD) profile illustrating the structural stability of the protein backbone (cyan) and ligand (magenta), showing minimal deviation and steady equilibration throughout the trajectory, indicative of a stable complex. (B) Root Mean Square Fluctuation (RMSF) profile showing residue-wise flexibility of the protein (cyan) and ligand (magenta). Moderate fluctuations are observed mainly in the loop regions, while key active-site residues maintain stability, confirming persistent and stable ligand binding during the simulation.

The root mean square deviation (RMSD) plot (Fig. 8A) showed that the backbone of COX-2 equilibrated to a stable level within the first 20 ns of simulation and remained uniformly at that value for the remainder of the 100 ns production run with small deviations. The protein backbone average RMSD equilibrated to 0.22 nm, with no evident structural drift or unfolding, and thus confirming the conformational stability of COX-2 in the presence of the ligand.

The ligand XGE-1 was also not very far from its initial docked conformation, with RMSD at all times being about 0.05–0.07 nm, which suggests that there is a stable and uniform binding pose throughout the simulation. This stable profile supports the high docking score and strong non-covalent interactions between the COX-2 binding site and XGE-1.

#### 5.6.2 RMSF analysis

The root mean square fluctuation (RMSF) analysis (Fig. 8B) was helpful in visualizing the flexibility of individual residues of COX-2 during the course of the simulation. A majority of the amino acid residues displayed fluctuations less than 0.20 nm, and slightly higher values were noted at the N-terminal and C-terminal ends, which are recognized flexible and solvent-exposed regions. Interestingly, residues contributing directly to the interaction with XGE-1, such as GLN289, HIS214, and LYS211, showed very low RMSF values (<0.10 nm) that indicate high local rigidity and strong ligand anchoring in the binding pocket. Low RMSF activity in the binding pocket is consistent with the solid ligand**–**protein interaction profile observed in the RMSD analysis.

### 5.7 Binding free energy

The MM/GBSA analysis (Table 7) revealed that XGE-1 exhibited the most favorable binding free energy with COX-2 (−36.3 ± 4.6 kcal/mol), followed by COX-1 (−24.0 ± 4.2 kcal/mol). The strong van der Waals (−46.8 kcal/mol) and electrostatic (−20.9 kcal/mol) contributions suggest tight hydrophobic packing and stable H-bonding within the COX-2 active site. In contrast, interactions with apoptotic regulators Bax and BCL were weaker (ΔG_bind >  − 15 kcal/mol), indicating lower affinity. These findings corroborate the in vitro anti-inflammatory and cytotoxic assays, underscoring COX-2 inhibition as the dominant mechanism of XGE-1.

**Table 7:** Binding Free Energy Analysis of Selected Protein-Ligand Complexes Using the MM/GBSA Method.

Protein	ΔG_bind (kcal/mol)
COX-1	−24.0 ± 4.2
COX-2	−36.3 ± 4.6
Bax	−13.6 ± 2.7
BCL	−12.0 ± 3.0
Casp9	−17.6 ± 3.4

## 6 Discussion

The current study underscores the pharmacological potential of X. granatum bark extracts, particularly the ethyl acetate fraction (EAF) and its isolated compound (+) catechin (XGE-1), by demonstrating strong antioxidant, anti-inflammatory, and cytotoxic properties. These results are aligned with and build upon prior investigations into the bioactivity of X. granatum and related mangrove species.

Antioxidant activity was most pronounced in the ethyl acetate fraction (EAF), which also exhibited the highest levels of total phenolics and flavonoids among all tested extracts. This trend is consistent with the reports of Dey et al. (2021) and Audah et al. (2022), who observed strong antioxidant activity in *X. granatum* leaf extracts enriched with polyphenolic compounds such as (+)-catechin, quercetin, and gallic acid 28, 29. The high radical-scavenging capacity observed in the present study therefore supports the hypothesis that antioxidant potential is primarily driven by the abundance of hydroxylated phenolic constituents capable of donating electrons and stabilizing free radicals. Comparable findings have also been documented in other mangrove species such as *Avicennia marina* and *Rhizophora mucronata*, reinforcing the conserved antioxidant mechanism across mangrove-derived polyphenols [30].

Similarly, the anti-inflammatory assays revealed pronounced membrane-stabilizing effects in both EAF and CME, indicating protection against heat- and hypotonicity-induced hemolysis. The isolated compound XGE-1 (identified as (+)-catechin) also exhibited substantial inhibition of RBC lysis, supporting its role as a key bioactive component. These findings align with earlier observations by Islam et al. (2019), who attributed the anti-inflammatory potential of *X. granatum* extracts to catechins and related phenolic derivatives [31]. The stabilization of erythrocyte membranes in this study suggests that catechin mediates anti-inflammatory action by preventing lysosomal membrane rupture and subsequent release of inflammatory mediators.

Moreover, the observed anti-inflammatory activity may be explained by the compound’s ability to stabilize cell membranes and prevent oxidative damage. Previous research on Camellia sinensis (+) catechins by Sekowski et al. (2018) showed similar protective mechanisms in erythrocyte membranes, indicating that flavonoids like (+) catechin modulate inflammatory responses through both antioxidant and membrane-stabilizing effects [32].

Cytotoxicity results from the brine shrimp lethality assay and MTT assay on MCF-7 breast cancer cells further support the therapeutic potential of EAF and XGE-1. The EAF demonstrated strong cytotoxicity (LC_50_ = 24.15 µg/ml), which falls within the “highly toxic” category (<100 µg/ml) per Hamidi et al. (2014). These findings are consistent with prior studies on X. granatum extracts. For instance, Darmadi et al. (2021) observed significant anticancer activity of ethyl acetate leaf extracts against HeLa and MCF-7 cells, attributed to high phenolic content [33].

XGE-1, identified as (+)-catechin through NMR analysis, exhibited clear dose-dependent antiproliferative activity against MCF-7 cells, confirming its cytotoxic potential. Although its efficacy was lower than that reported for epigallocatechin gallate (EGCG) and epicatechin gallate (ECG) from green tea, the observed activity remains noteworthy given the natural origin and structural simplicity of (+)-catechin isolated from *X. granatum*. Differences in potency may stem from variations in molecular conformation, purity, or the absence of synergistic interactions present in the crude extract.

The molecular docking analysis provided further insight into the dual mechanism of XGE-1. The compound displayed strong binding affinities toward COX-2 and Caspase-9, key targets in inflammation and apoptosis, respectively. These findings support the hypothesis that (+)-catechin contributes to both anti-inflammatory and pro-apoptotic effects through simultaneous modulation of these pathways. Comparable dual-target interactions of catechin derivatives have been reported by More-Adate et al. (2024) [34], reinforcing the concept of catechins as multifunctional agents with potential applications in chemoprevention and inflammation control.

The binding interactions revealed hydrogen bonds, π-alkyl, and van der Waals contacts with amino acid residues within the active sites of COX-2 and Caspase 9. These findings align with the pharmacophoric features required for inhibition of these enzymes and support the in vitro anti-inflammatory and cytotoxic activities observed. While (+)-catechin displayed favorable binding energies and stable molecular interactions with COX-2 and Caspase-9, certain pharmacokinetic limitations restrict its direct therapeutic translation. Catechins generally suffer from poor oral bioavailability, extensive metabolism, and rapid clearance, which may reduce their in vivo efficacy. Structural modification, nanoformulation, or combination therapy could enhance their pharmacological potential [35]. Acknowledging these limitations emphasizes that, while promising, XGE-1 represents a preliminary lead that warrants further optimization and preclinical validation.

To further validate the structural stability of the docked complexes, molecular dynamics (MD) simulations were conducted on the XGE-1–COX-2 complex for 100 ns. The root mean square deviation (RMSD) analysis demonstrated that the protein backbone reached stability after \sim 20 ns, maintaining a consistent RMSD of ∼ 0.22  nm throughout the simulation. The ligand XGE-1 also remained stable within the binding pocket, with an RMSD of \sim 0.05  nm, suggesting a strong and sustained interaction. Additionally, root mean square fluctuation (RMSF) analysis indicated limited residue flexibility, primarily at the terminal regions, while the active site residues interacting with XGE-1 remained stable. These results confirm the thermodynamic stability and favorable binding conformation of the complex, supporting the potential of XGE-1 as a stable COX-2 inhibitor.

The energetic decomposition revealed that van der Waals and electrostatic interactions were the major contributors to complex stability, whereas polar solvation energy opposed binding, as typically observed in hydrophobic pocket interactions [36]. Persistent hydrogen bonding and low RMSD fluctuations during the 100-ns MD trajectory further confirmed the structural integrity of the COX-2–XGE-1 complex. In contrast, the relatively less favorable ΔG_bind values for Caspase-9 and Bax indicate moderate affinity, suggesting that XGE-1 might modulate apoptotic signaling through secondary interactions with these proteins, consistent with the observed cytotoxic and pro-apoptotic activity in cancer cell assays.

Overall, the combined molecular docking, MD simulation, and MM/PBSA results strongly support the dual anti-inflammatory and anticancer potential of XGE-1. The computational findings align with experimental outcomes, strengthening the mechanistic evidence that the phytochemicals in *X. granatum* bark exert their biological effects through stable and energetically favorable interactions with inflammation- and apoptosis-related proteins.

In summary, our findings reinforce and expand the existing body of literature by providing compound-level evidence for the bioactivity of bark extracts. The isolation and functional validation of (+) catechin adds to the phytochemical profile of this species and supports its traditional medicinal use. While similar compounds have been studied in other plants, this is among the first studies to report (+) catechin from the bark of X. granatum with corroborating in vitro and *in silico* evidence for its pharmacological relevance.

Future studies should focus on evaluating the pharmacokinetics, in vivo efficacy, structure–activity relationship (SAR) studies to fully harness its therapeutic potential and formulation strategies of (+)-catechin to facilitate its translation into preclinical and eventually clinical applications.

Declarations

## Ethics approval and consent to participate

Not applicable. This study did not involve human participants or animal experiments requiring ethics approval.

Consent for publication

Not applicable. This manuscript does not contain any individual person’s data.

Availability of data and material

The datasets analyzed during the current study are available from the corresponding author on reasonable request.

## Funding

Not available
